# BRS Carmem Grape Liqueurs: Influence of Alcoholic Base on Physicochemical Characteristics, Anthocyanin Composition, and Sensory Acceptance

**DOI:** 10.3390/molecules30112270

**Published:** 2025-05-22

**Authors:** Francielli Brondani da Silva, Taís Gaspar, Victoria Diniz Shimizu-Marin, Yara Paula Nishiyama-Hortense, José Pérez-Navarro, Sergio Gómez-Alonso, Ellen Silva Lago-Vanzela

**Affiliations:** 1 São Paulo State University (UNESP), Institute of Biosciences, Humanities and Exact Sciences, Cristóvão Colombo 2265, São José do Rio Preto 15054-000, Brazil; francielli.brondani@unesp.br (F.B.d.S.); tais.gaspar@unesp.br (T.G.); victoria.shimizu@unesp.br (V.D.S.-M.); yara.nishiyama@unesp.br (Y.P.N.-H.); 2 Regional Institute for Applied Scientific Research (IRICA), University of Castilla-La Mancha, Avda. Camilo José Cela 1B, 13071 Ciudad Real, Spain; jose.pnavarro@uclm.es (J.P.-N.); sergio.gomez@uclm.es (S.G.-A.); 3 Higher Technical School of Agronomic Engineering, University of Castilla-La Mancha, Ronda de Calatrava 7, 13071 Ciudad Real, Spain; 4 Faculty of Chemical Sciences and Technologies, University of Castilla-La Mancha, Avda. Camilo José Cela 10, 13071 Ciudad Real, Spain

**Keywords:** Brazilian grape, social technology, anthocyanins, HPLC-DAD-ESI-MS/MS

## Abstract

Grape liqueurs are a promising approach to diversifying fruit-derived beverages and adding value to local raw materials. This study evaluated the impact of cereal alcohol (A) and white cachaça (C) on the physicochemical composition, anthocyanin profile, and sensory attributes of liqueurs made with the Brazilian cultivar BRS Carmem. Both products met regulatory requirements (alcohol content > 15% *v*/*v* and sugar > 100 g⋅L^−1^). The alcoholic base significantly influenced most physicochemical parameters but not the anthocyanin profile. The liqueur with A resulted in higher extraction of organic acids (0.39 vs. 0.33 g tartaric acid⋅100 g^−1^) and phenolic compounds (607.45 vs. 457.64 mg gallic acid⋅100 g^−1^). HPLC-DAD-ESI-MS/MS analysis showed a predominance of diglycosylated anthocyanins (98%), with concentrations of 420.04 mg⋅L^−1^ (A) and 456.44 mg⋅L^−1^ (C). Both liqueurs were well accepted (overall impression: A = 7.1, C = 7.2) with good purchase intent (A = 63.03% and C = 56.75%). Significant differences were observed for appearance and color (preferred in A) and aroma (preferred in C). These attributes correlated strongly with the overall impression, but flavor and alcohol content were the key factors influencing purchase decisions. The findings demonstrate that the choice of alcoholic base affects the composition and sensory acceptance of grape liqueurs, highlighting their importance to enhance the product’s quality.

## 1. Introduction

The global consumption of alcoholic beverages remains significant, particularly in emerging markets, where younger, expanding middle classes, increasing urbanization, and a rising interest in premium and innovative alcoholic beverages are driving growth. With a diverse range of offerings catering to different consumer preferences, the International Wine and Spirit Research highlights promising markets of scotch, sparkling wine, and liqueurs [1]. Consumers are also increasingly attracted to products that emphasize the origin of their ingredients and the history behind their production, especially those linked to family farming. Considering the simplicity of production, the liqueur market presents an opportunity to blend tradition and modernity by fostering innovation and promoting the sustainable use of local resources. Moreover, contemporary consumers are prioritizing healthier and more sustainable choices, favoring beverages that strike a balance between moderation, responsibility, and sensory appeal [2,3,4,5].

With a balanced sweetness profile, intense purple color (with pigments in the skin and pulp), and distinct fruity notes [6], the BRS Carmem grape (Muscat Belly A × BRS Rúbea) offers significant potential liqueur production, which requires ingredients with well-defined and distinctive flavors. Furthermore, BRS Carmem shows good adaptability to various climatic and soil conditions, supporting its cultivation in different regions of Brazil, as well as a complex and interesting concentration of bioactive compounds, such as phenolic compounds, even when immature [7]. Therefore, the use of this cultivar in such products can represent an opportunity to explore and value local fruits, aligning with the growing demand for products that emphasize the origin and tradition of ingredients.

However, although the Brazilian Agricultural Research Corporation (Embrapa) developed this cultivar as an alternative for processing [8], research on its potential for liqueur production is scarce. In general, grape-based beverages are predominantly represented by wines and juices, which have long-standing market acceptance and well-established production processes. As an example, the phenolic composition of juices and wines from the BRS Carmem grape has been evaluated in previous studies [9,10,11]; however, in the current literature, there are no reports on BRS Carmem liqueur. This is of utmost importance, as phenolic compounds (PCs) may be closely related to sensory characteristics and, particularly, to the color of the final product.

PCs are mainly extracted during the maceration phase of production and can be influenced by numerous factors such as time, type and concentration of alcohol, temperature, and the ratio of fruit to extracting solution [12]. Among these factors, the choice of alcoholic source is particularly relevant, as it directly influences the extraction of bioactive compounds and the sensory characteristics of the liqueur [3,13]. In Brazil, artisanal liqueurs are commonly produced using cachaça—the typical and exclusive denomination for Brazilian sugarcane spirits with an alcohol content ranging from 38% to 48% (*v*/*v*) [14]—especially the unaged and colorless one known as white cachaça (C). Its wide availability and low cost make cachaça a frequent choice among small producers [3], while also representing a culturally and economically significant local product [15,16]. Beyond these aspects, cachaça contributes distinctive sensory attributes, such as body, aroma, and flavor, resulting from esters, phenolics, and volatile compounds derived from fermentation and distillation [17]. However, its vast artisanal production often lacks standardization and quality control [15,17]. High-quality C requires strict control of various parameters, including the raw material, yeast strain, fermentation and distillation processes, acidity, and the levels of copper, higher alcohols, aldehydes, ethyl carbamate, and methanol [16]. In contrast, neutral alcohols—particularly cereal alcohol (A), which is highly refined and odorless—are commonly recommended for liqueur production, as they allow the fruit’s sensory attributes, such as aroma and flavor, to stand out without interference from the alcoholic base [3,18].

Dechkunchorn et al. [13] demonstrated that a 60% ethanol solution led to a higher total phenolic content in the final product than a 40% solution, underscoring the impact of alcohol concentration on compound extraction. Similarly, Neves et al. [3] reported that the use of C instead of cereal alcohol A in jabuticaba liqueurs modified the volatile and anthocyanin profiles. Liqueurs made with C presented higher variety and amounts of terpenes and volatile phenolic compounds, which are desirable due to their positive impact on aroma and antioxidant capacity. Regarding color, these liqueurs showed higher hue values and increased color intensity, indicating a shift toward a yellow-orange coloration. These effects were associated with the presence of copper in the C, which complexed with anthocyanins and resulted in a greater degree of anthocyanin polymerization, highlighting that the composition of the alcoholic matrix can significantly influence both the chemical composition and the product’s color.

It is also important to highlight that, although grape-based liqueurs have the potential to diversify the portfolio of grape-derived products, their development remains relatively underexplored compared to wines and juices. Among the primary attributes influencing the acceptance and characterization of these products, color plays a central role, being directly affected by the presence and stability of PCs, particularly anthocyanins. These compounds not only contribute to the visual quality of liqueurs but also to perceived consumer value and the potential bioactivity of the final product. In this context, the present study aimed to develop a grape liqueur using the Brazilian cultivar BRS Carmem, evaluating the influence of two different alcoholic bases—A and C—on anthocyanin composition, as well as on the physicochemical and sensory properties of the liqueurs. This comparison is especially relevant considering that the choice of alcoholic matrix can significantly affect the extraction, stability, and interactions of PCs, leading to notable differences in product color. The use of C, for instance, may promote reactions that alter hue and color intensity due to the presence of trace metal, such as copper and other constituents that favor anthocyanin polymerization. In contrast, A, due to its neutral character, tends to preserve the original pigment profile of the fruit. By emphasizing the characteristics of liqueurs produced with these two alcoholic bases, this study provides insights for the development of visually appealing products that valorize national cultivars.

## 2. Results and Discussion

### 2.1. Physicochemical Characteristics

**Figure 1:**
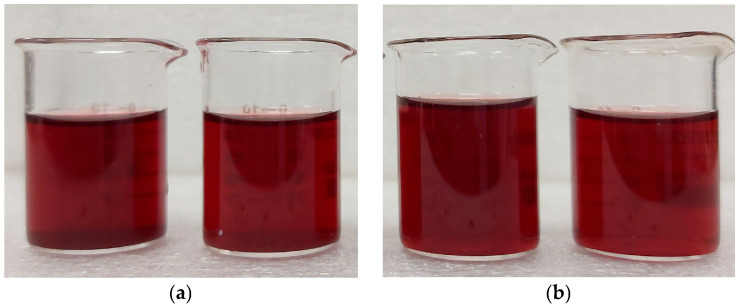
Visual appearance of grape liqueurs from BRS Carmem (**a**) produced with cereal alcohol (two biological replicates) and (**b**) produced with white cachaça (two biological replicates).

The liqueurs produced (Figure 1) had an average yield of approximately 65%. While this yield is lower than the values reported by Oliveira et al. [4] for soursop liqueurs (from 79.29% to 85.25%), it is important to note that their formulations were based on fruit pulp. In contrast, the present study utilizes whole grapes, which naturally results in co-products such as pomace, including peels and seeds. Yield is a key parameter for assessing processing feasibility and potential commercialization; however, studies addressing this aspect remain scarce in the literature.

Table 1 presents the physicochemical characterization results of both liqueurs. The soluble solids (SS) content of liqueurs varies depending on the ingredients used, particularly the sugar or syrup content and the type of fruit employed [19]. Despite these variations, SS levels play a crucial role in defining the sensory characteristics of liqueurs, especially their sweetness, which is a key attribute appreciated by consumers.

In this study, the SS values of the developed liqueurs are within the range reported in the literature for fruit liqueurs. Among the liqueurs studied, liqueur with A exhibited higher SS values than liqueur C. The higher SS content in the liqueur with A can be attributed to the specific chemical composition of the alcoholic base, which may enhance the solubilization and extraction of sugars from the fruit. Nascimento et al. [20] found values between 30.5 and 31.73 °Brix for banana liqueurs prepared with different concentrations of cinnamon powder and bark, while Oliveira et al. [21] reported lower values (14.33–16.38 °Brix) for *Anacardium humile* liqueur. Similarly, Leonarski et al. [19] observed SS values ranging from 15.8 to 38.9 °Brix in different formulations of jabuticaba, blackberry, and guabiroba liqueurs. These findings confirm that the SS content of both BRS Carmem grape liqueurs, even with the significant differences, is consistent with those reported for fruit liqueurs, reinforcing their potential to meet consumer expectations regarding sweetness and overall sensory quality [19].

The total sugar content followed the same trend as the SS, being higher in the liqueurs made with A. However, for both samples the total sugar complies with current Brazilian legislation [14], which establishes a sugar content range of 100 g⋅L^−1^ to 350 g⋅L^−1^, classifying them as sweet or fine liqueurs. These values also align with the Code of Federal Regulations (CFR) of the United States, which requires a minimum sugar content of 2.5% by volume for liqueurs and cordials [22], and with the European Parliament and the Council of the European Union, which sets a minimum total sugar content of 100 g⋅L^−1^ [23].

The pH and total acidity (TA) parameters of the evaluated liqueurs also showed statistically significant differences (*p* ≤ 0.05). The liqueur made with A exhibited higher acidity and, consequently, lower pH values. This result suggests that the alcohol source used in the preparation may have influenced these parameters likely due to the presence of different levels of organic acids and their interactions with the alcohol during the extraction process. The higher acidity observed in the liqueur with A could be related to the interaction between the alcohol and the grape’s organic acids, which may have resulted in greater extraction of these compounds. However, it is important to note that the difference between the mean values was relatively small, particularly for pH (approximately 3.4%). The values obtained in the present study indicated greater acidity compared to fruit liqueurs reported by other authors, such as *Anacardium humile* liqueur (pH = 3.53–3.71; acidity = 0.04–0.14% acetic acid) [21] and banana liqueur with cinnamon (pH = 4.65–4.87; acidity = 1.35–1.42 g⋅L^−1^ citric acid) [20]. Such differences are expected since different fruits and ingredients were used. Nevertheless, it is important to consider the relevance of this parameter to the sensory quality of the product. According to Matias-Guiu et al. [24], spirits typically have a pH between 2.8 and 5, and a lower pH can diminish pungent and fruity aromas.

The total phenolic compounds (TPC) value was higher in the liqueur produced with A than in the one made with C. Nevertheless, both alcoholic bases promoted a greater extraction of phenolic compounds compared to values reported by other authors for different fruit liqueurs. Leonarski et al. [19] found that jabuticaba liqueur had the highest TPC values (84.15 mg GAE⋅100 g^−1^), followed by guabiroba (34.91 mg GAE⋅100 g^−1^) and blackberry (13.80 mg GAE⋅100 g^−1^). These results highlight the potential of grapes, particularly the BRS Carmem variety, as a valuable raw material for liqueur production, contributing to elevated PC concentrations and, consequently, to the functional appeal of the final product. It is known that the amount of TPC depends on the type of raw material, the degree of fruit ripeness, soil type, climate, cultivation conditions, solar exposure, and post-harvest storage, as well as genetic characteristics, natural selection, or plant breeding programs [25,26]. Additionally, it is important to highlight that the PCs present in fruits directly influence the color of the produced liqueurs. Color is a psychological attribute often decisive in selecting comparable food products [27]. Red, in particular, is commonly associated with ripeness, superior flavor, and other pleasant characteristics of plant-based products, including liqueurs [28].

It is noteworthy that, even when macerating the grapes with different alcoholic sources but with the same alcohol content, such as A or C in the present study, the alcoholic base may influence the extraction of the compounds in the grapes. This is likely due to A being a purer matrix, with a neutral taste and aroma. In contrast, C contains secondary compounds derived from the fermentation and distillation of sugarcane juice, such as higher alcohols, esters, and aldehydes, known as congeners [16,29]. These compounds can interact with those extracted from the fruit, such as sugars, acids, and PCs, modifying the physicochemical parameters of the liqueur, including pH, acidity, solubility, and stability of phenolic and volatile compounds, which directly impact the product’s final characteristics.

The color characteristics of the grape liqueurs were significantly affected (*p* ≤ 0.05) by the alcoholic source. Liqueurs produced with A exhibited a lower L* value, indicating a darker color than those made with C. Additionally, liqueur A displayed a more saturated color and, although both liqueurs (A and C) showed an orange hue, the *h°* value determined for liqueur A suggested a hue closer to red. Similarly, Neves et al. [3] observed a hue measurement indicating the development of an orange-toned color in jabuticaba liqueurs made with both A and C which, according to the authors, is associated with beverage aging. This color change may result from anthocyanin polymerization and the consequent bathochromic effect [30]. In their study, the liqueur made from C exhibited a higher hue value compared to the one produced with cereal alcohol, as observed in the present study. The authors attributed this phenomenon to the presence of copper in C, as heavy metals have a high complexation capacity with anthocyanins, contributing to the bathochromic effect. They also reported a higher degree of anthocyanin polymerization in the C-based liqueur, which may explain the formation of more intense yellowish tones. Although copper was not identified in the samples analyzed in this study, C may contain trace amounts of such elements, potentially leading to a similar effect.

Several researchers have proposed color indices that allow a direct correlation with the visual appearance of specific food products. These indices are characterized by a strong association with the external visual color of fruits [31]. In the present study, the Color Index for Red Grapes (CIRG) was also calculated, a parameter commonly used to categorize the coloration of red grapes [32,33] and their derived products, such as grape juice [34]. CIRG enables an objective definition of external color in different red grape cultivars, classifying berries into five categories: green-yellow (CIRG < 2); pink (2 < CIRG < 4); red (4 < CIRG < 5); dark red (5 < CIRG < 6); and blue-black (CIRG > 6) [33]. Despite the differences highlighted by the colorimetric parameters (L*, C*, and *h°*), both liqueurs analyzed in this study obtained CIRG values that categorize them as pink—2.16 for liqueur A and 2.12 for liqueur C. Further sensory analysis is recommended to determine whether color differences are perceptible and influence consumer acceptance.

The alcohol content of the produced liqueurs complies with current Brazilian legislation [14], which establishes a range between 15% and 54% at 20 °C. These values also align with the regulations of the United States, which require a minimum alcohol content of 15% for liqueurs and cordials [22], and with the European Parliament and the Council of the European Union, which set the same minimum threshold [23]. According to Vieira et al. [35], most industrial fruit liqueurs have a labeled alcohol content between 18% and 25% (*v*/*v*), with a consumer preference for sweet liqueurs. Additionally, contaminant levels were below the limits established by national regulations, which set thresholds of 200 mg⋅100 mL^−1^ for methyl alcohol, 5 mg⋅kg^−1^ for copper, and 0.2 mg⋅L^−1^ for lead [14].

### 2.2. Identification and Quantification of Anthocyanins in the Liqueur

To evaluate the liqueurs’ potential as a source of anthocyanins, a qualitative and quantitative characterization of these compounds was conducted. A total of 13 anthocyanins were identified (Table 2), derived from the main anthocyanidins commonly found in grapes: delphinidin (dp), cyanidin (cy), petunidin (pt), peonidin (pn), and malvidin (mv), in both their monoglycosylated (3-glc) and diglycosylated (3,5-glc) forms.

Additionally, some anthocyanins were detected in their acylated forms, specifically caffeoylated (cfglc) and *p*-coumaroylated (cmglc) derivatives. In both liqueurs, a predominance of diglycosylated anthocyanins was observed, accounting for from 97.4% to 97.91% of the total. Nishiyama-Hortense et al. [7] also reported the predominance of these anthocyanins in BRS Carmem grapes from different harvests, but with a (3,5-diglc/3-glc) ratio ranging from 2.22 to 5.64, which is significantly lower than the values observed in the liqueurs of the present study. This even greater predominance of diglycosylated anthocyanins may be attributed to the enhanced stability conferred by the presence of two sugar molecules bound to anthocyanidins. This suggests that, although anthocyanins were extracted during the maturation period, degradation reactions likely occurred due to oxygen exposure. As a result, only the most stable anthocyanins remained in the final liqueur composition.

Nishiyama-Hortense et al. [7] also reported the predominance of anthocyanins derived from dp, which does not corroborate the findings of the present study. In both liqueurs, anthocyanins derived from mv were the most abundant, with the diglycosylated form being the predominant one (36.03–38.36%), followed by the diglycosylated coumarylated form (31.89–33.81%). Among the monoglycosylated anthocyanins, which were present in lower amounts, mv-3-glc showed the highest percentages (1.13–1.27%). After mv, pn-derived anthocyanins were the most abundant (13.73–13.82%). Both mv and pn are methylated anthocyanins, and the presence of a methoxyl group contributes to the structural stability of these molecules, making them more resistant to degradation processes compared to non-methylated anthocyanins. Neves et al. [3] also observed this stability of pn in jabuticaba liqueurs produced with cereal alcohol and cachaça.

**Figure 2:**
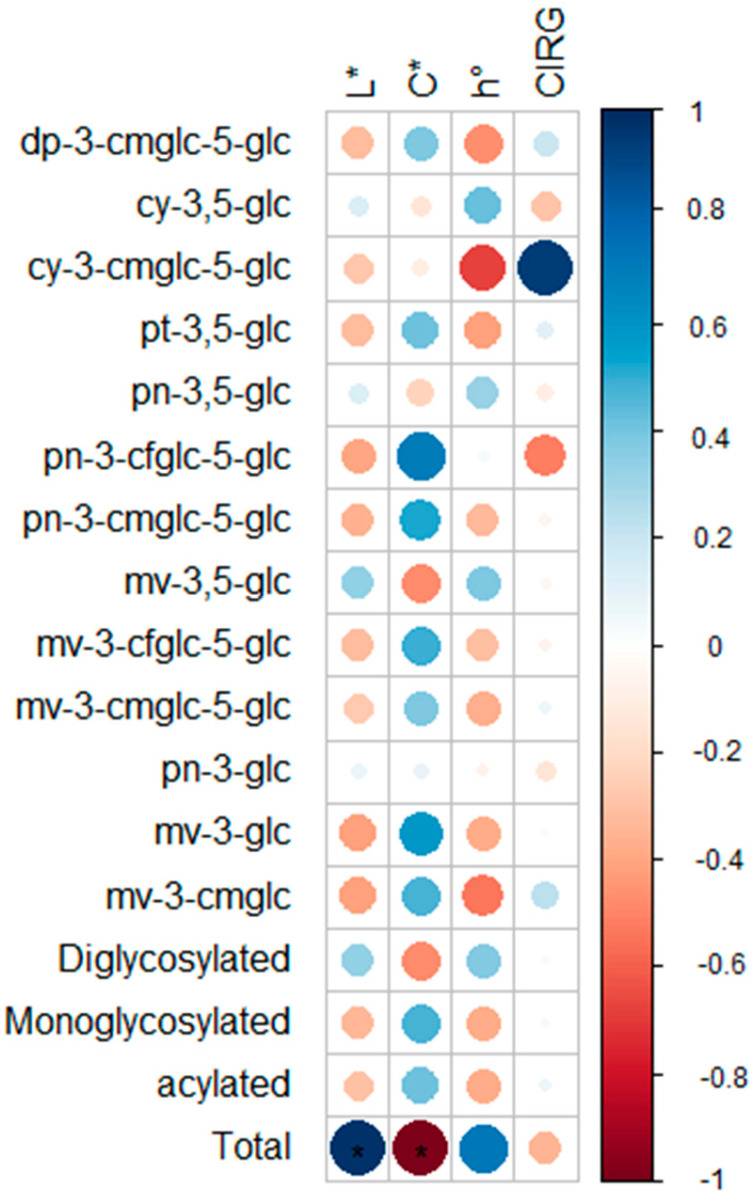
Pearson correlation between individual anthocyanins and color parameters in BRS Carmem grape liqueurs. *: *p* ≤ 0.05. The size of the circles indicates the strength of the correlations, with larger circles representing stronger correlations.

It is also important to highlight the presence of the acylated anthocyanins. These compounds are known for their enhanced resistance to degradation and their contribution to long-term color retention in grape-derived products. The diversity and complexity of the anthocyanin profile in BRS Carmem grapes, including a high proportion of more stable forms such as diglycosylated and methoxylated anthocyanins, underscore the potential of this cultivar for processing applications, including liqueur production. This is particularly relevant not only for the functional attributes of the final product, but also for its sensory quality, as anthocyanins are key contributors to color stability and visual appeal. Xie et al. [36] reported that the higher levels of acylated anthocyanins in “Petit Verdot” grapes enhance the color stability of the wine, since they have increased chemical stability. Color is one of the main quality characteristics of liqueurs, determined by the quantity, type, and relative proportions of anthocyanins present. Several authors have already associated specific anthocyanins with different contributions to the final product’s color [34,37,38,39]. To elucidate the contribution of anthocyanin compounds to color in the liqueurs of this study, the Pearson correlation analysis was performed to investigate the relationship between color parameters (L*, C*, *h*°, and CIRG) and the individual anthocyanins identified (Figure 2).

No significant correlation was observed between any individual anthocyanin and the color parameters in the BRS Carmem liqueurs (*p* > 0.05). This result suggests that the chromatic attributes of the liqueurs were not directly influenced by the concentration of a single anthocyanin compound, but rather by the combined effect of multiple pigments and possibly their interactions or polymerization during processing. Several authors have reported the importance of polymeric anthocyanins in the coloration of grape-derived products, particularly wine [38,39,40,41]. Li et al. [40] reported that the color intensity of blueberry wine was positively associated with co-pigmentation between anthocyanins and phenolic acids or flavanols, which increased during storage due to the formation of more stable pigments. Similarly, Shi et al. [42] reported that the intensity of wine color depends more on the concentration of anthocyanin derivatives than on monomeric anthocyanins. Such pigments are formed through direct polymerization between anthocyanins and flavan-3-ols or proanthocyanidins, as well as through “bridge-mediated” polymerization involving anthocyanins themselves or in combination with proanthocyanidins or flavan-3-ols. Casassa et al. [39] also highlighted the significance of pyranoanthocyanins formed by covalent reactions between monomeric anthocyanins and pyruvic acid or acetaldehyde. These compounds may be formed during maceration or aging and, due to their bathochromic shift, can confer orange to orange-brown hues. Their relatively lower molar extinction coefficients, when compared to intact anthocyanins, may contribute to lower C* and hue characteristics typical of certain wines [38,39]. Regarding the liqueurs, it should be noted that the complexity of compounds present is lower than that observed in wine, which may have led to less pronounced effects on coloration. This factor may have influenced the observed results, as pigment formation and interactions in liqueur, although relevant, are less complex compared to those occurring during the winemaking process. It is also worth noting that the absence of significant correlations may be attributed to the similarity among the samples, which showed no substantial differences in their anthocyanin profiles or color attributes. Furthermore, the predominant anthocyanins identified in the liqueurs—derivatives of mv and pn—may contribute to similar color tones across samples. Casassa et al. [38] reported that *O*-methylation of anthocyanidin aglycones produces a slight reddening effect, with malvidin generally associated with red hues and peonidin contributing more toward magenta tones [43].

Interestingly, a significant positive correlation (*p* < 0.05) was observed between total anthocyanin content and the L* value, indicating that liqueurs with higher anthocyanin concentrations exhibited lighter color. Conversely, a significant strong negative correlation was found with the C* parameter, suggesting a decrease in color saturation as anthocyanin levels increased. Although this may seem counterintuitive—since anthocyanins are generally associated with darker and more intense colors—such behavior can be explained by the complex interactions that anthocyanins undergo in hydroalcoholic systems, including co-pigmentation, degradation, and polymerization. The presence of different anthocyanin structures may result in higher total content without necessarily enhancing color intensity. The blending of multiple anthocyanin tones might lead to a less vivid overall appearance, increasing L* while reducing C*.

It is important to highlight that several factors directly influence the final coloration of grape liqueurs. In addition to anthocyanin structure and concentration, other variables such as storage temperature, pH, oxygen availability, and the presence of co-pigments, ascorbic acid, sugars, and their degradation products can also affect color stability [43,44]. The absence of significant correlations reinforces the complexity of color expression in grape-based products, where mechanisms such as co-pigmentation, acylation, oxidation, and the overall matrix composition may play a more decisive role than the concentration of individual pigments alone.

### 2.3. Sensory Acceptance

The results obtained from the sensory acceptance data analysis are shown in Table 3. A total of 111 liqueur consumers—50 males and 61 females, with an average age of 24 and ranging from 18 to 68 years—participated in the study. All participants were Brazilian and either students or members of the institution where the analysis was conducted. Evaluators were considered liqueur consumers only if they had indicated in a preliminary questionnaire that they liked liqueurs (i.e., selected “like a lot” or “like a little” on a five-point hedonic scale) or if they reported consuming liqueurs at least once every six months. The sensory analysis of the liqueurs revealed how consumers evaluated different attributes and their influence on the final preference.

No statistically significant differences (*p* > 0.05) were observed in the attributes: consistency, flavor, sweetness, alcohol content, and overall impression. The overall impression results indicated that both liqueurs were well accepted by the evaluators, which may suggest that both alcohol sources were well-received. Regarding the aroma, the formulation containing C showed a higher preference among consumers (*p* ≤ 0.05). This result may be associated with the presence of characteristic aromatic compounds resulting from the production process of C. Neves et al. [3], in a study on jabuticaba liqueurs, reported that the liquor made from C exhibited greater aromatic complexity when compared to A, including terpenes and volatile phenolic compounds. To confirm the contribution of the alcoholic base to the aroma profile of the liqueurs, it is recommended that future studies include the identification and quantification of volatile compounds using gas chromatography coupled to mass spectrometry (GC-MS) or other appropriate analytical techniques. For the attributes “appearance” and “color”, the formulation made with A had higher acceptance. Although no significant difference was observed in anthocyanin content, the liqueurs exhibited distinct chromatic parameters (Section 2.1), which were likely perceived by consumers. This suggests a preference for the sample with a lighter color (lower L* value) and greater saturation (C*), indicating a stronger tendency toward a more vivid red hue.

**Figure 3:**
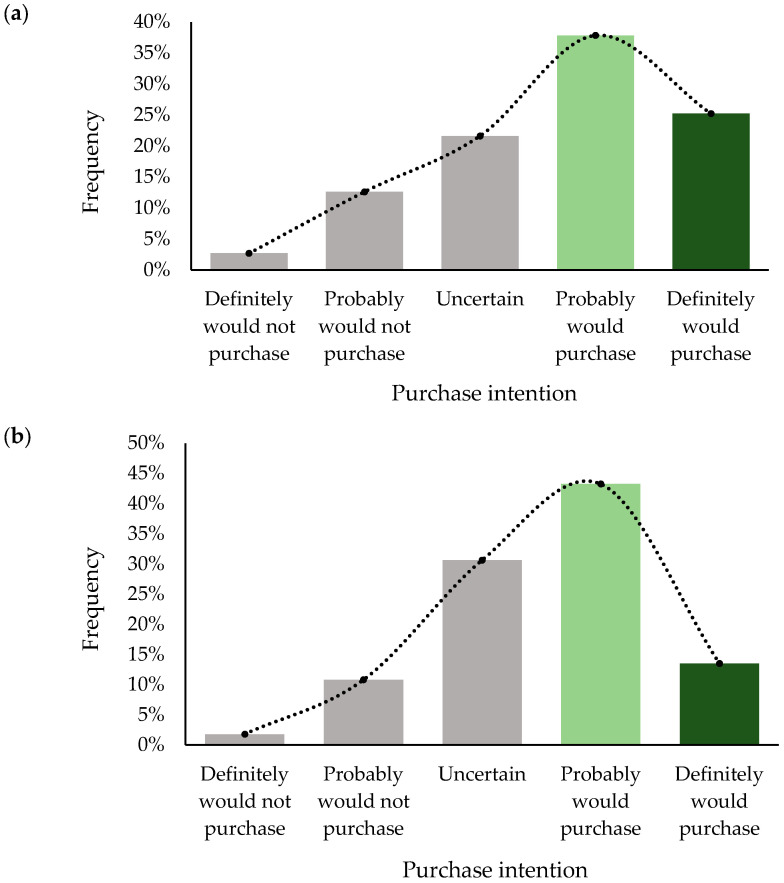
Frequency distribution of purchase intention for BRS Carmem grape liqueurs. (**a**) Liqueur produced with cereal alcohol and (**b**) liqueur produced with cachaça. Green bars highlight the responses indicating a positive purchase intention.

The purchase intention rate, defined as the frequency of responses indicating positive purchase intent, was 63.03% for the formulation with C and 56.75% for A. Although both liqueurs were well accepted, the frequency distribution of purchase intention (Figure 3) reveals differences between the formulations. The curve for the liqueur made with C is more skewed to the left (skewness = −0.57, against—0.42 for A), suggesting a higher purchase intention for these samples. The samples with A also received higher scores, indicating uncertainty in the purchase decision (31% against 22% for C samples), which may reflect greater hesitation among consumers when evaluating these samples.

**Figure 4:**
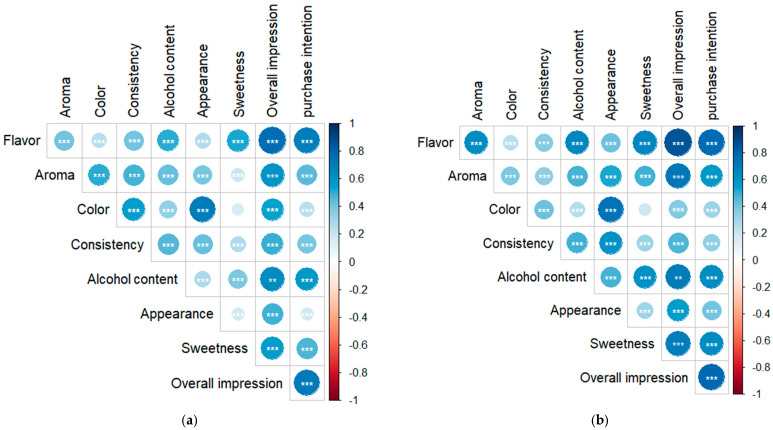
Pearson correlation for the sensory attributes of BRS Carmem grape liqueurs. (**a**) Liqueur produced with cereal alcohol and (**b**) liqueur produced with cachaça. **: *p* < 0.01; ***: *p* < 0.001. The size of the circles indicates the strength of the correlations, with larger circles representing stronger correlations.

The Pearson correlation analysis for the sensory acceptance and purchase intention results is shown in Figure 4. The analysis showed that, for both samples, all the analyzed parameters presented a significant correlation (*p* ≤ 0.05) with each other, except between sweetness and color.

Notably, for both samples, the overall impression exhibited the highest correlations with flavor, alcohol content, and sweetness. Interestingly, for the C sample, aroma also showed a strong correlation. A similar behavior can also be observed for purchase intention, likely due to its strong and significant correlation with overall impression. Thus, the preference for the aroma of the liqueur made with C may have contributed to the higher positive purchase intention for these samples.

Rodrigues et al. [45] reported that curiosity about the taste of a new product is an important factor that leads consumers to try a new beverage. These authors also concluded that the price of the alcoholic beverage was not a relevant factor for consumers in trying these new beverages. The results obtained in this study corroborate those of Neves et al. [3], where A and C were used to formulate jabuticaba liqueurs, and the sensory analysis showed that the two liqueurs obtained were considered similar by consumers.

## 3. Materials and Methods

### 3.1. Reagents and Standards

All solutions were made of deionized water (18.2 MΩ cm, Milli-Q, Millipore, Bedford, MA, USA). All solvents were HPLC-grade, and all chemicals were analytical grade (99%). The standard used for methanol determination was methyl-4-pentanol-2, while those used for anthocyanin analyses were the diglucoside (3,5-glc) derivatives of malvidin (mv) and cyanidin (cy), from Sigma-Aldrich (Madrid, Spain), peonidin (pn) from Phytolab (Vestenbergsgreuth, Germany), the monoglucoside (3-glc) derivatives of pn and cy, purchased from Sigma-Aldrich (Spain), and mv (Extrasynthese, Genay, France).

### 3.2. Grapes and Ingredients for Liqueur Production

The BRS Carmem grape variety was acquired from the Advanced Center for Research on Rubber Trees and Agroforestry Systems of the Agronomic Institute (IAC), located in the municipality of Votuporanga, State of São Paulo, Brazil (20°20′ S and 49°58′ W, altitude 525 m above sea level). Grapes were cultivated on the IAC 572 rootstock and trained using the trellis system. After harvest, the grapes were transported to the Fruit and Vegetable Processing Laboratory of the Department of Food Engineering and Technology at the São Paulo State University, Institute of Biosciences, Humanities and Exact Sciences, São José do Rio Preto, Brazil. Each bunch had its berries detached, and those with imperfections or signs of rot were discarded. The berries were then washed with potable water and sanitized through immersion in a chlorinated solution (0.005% *v*/*v* active chlorine) for 20 min. After sanitization, the grapes were rinsed with potable water, placed on paper towels, and left on a previously sanitized stainless steel countertop with 70% alcohol to remove excess water. Subsequently, they were stored in food-grade plastic bags and kept below freezing (−18 °C) until the start of analyses or processing, respectively.

For the liqueur processing, C (~42°), sourced from the JP distillery in Itupeva, São Paulo, and A (~94 °ABV), obtained from the local market in São José do Rio Preto, São Paulo, were used as alcoholic bases. Inverted sugar from the local market was used to sweeten the liqueurs. Before production, A was diluted with potable water to match the alcohol content of C.

### 3.3. Liqueurs Production

**Figure 5:**
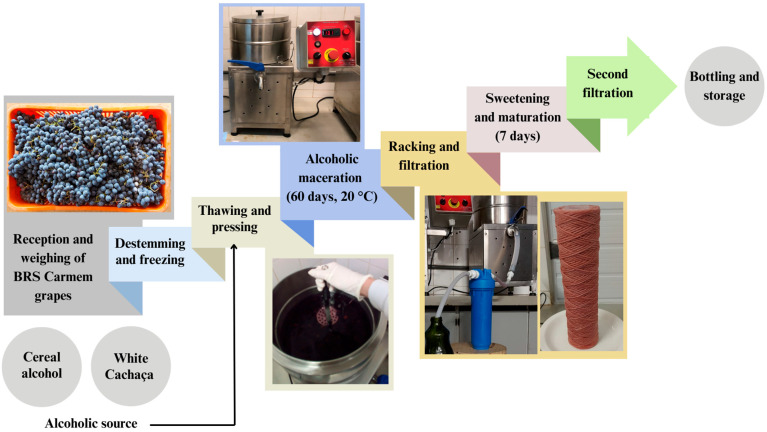
Flowchart of the liqueur production process from BRS Carmem grapes.

The production of the liqueurs using the two alcoholic sources, A and C, followed a formulation adapted from EMBRAPA [46] and was conducted in accordance with Good Manufacturing Practices [47]. The flowchart of the process can be observed in Figure 5. The previously frozen grapes were added to 10 L stainless steel tanks (INOXTECH, Indaiatuba, Brazil) under controlled temperature (20 ± 3 °C) with the respective alcoholic source in a 1:0.5 (*w*/*w*) ratio. For this step, A was pre-diluted with potable water to obtain a hydroalcoholic solution with the same alcohol percentage as C (~42°). The production was performed in duplicate, with two independent tanks per treatment—two tanks with grapes and source A, and two with grapes and source C—thus yielding two biological replicates for each type of liqueur. After thawing, the grapes were pressed using a stainless steel utensil and left to macerate.

The maceration period adopted was 60 days, determined based on preliminary tests comparing durations of 15, 30, and 60 days. These initial trials indicated that 60 days provided the highest extraction of phenolic compounds and more pronounced color intensity in the liqueurs. Given this study’s focus on the phenolic composition and its influence on color, this duration was considered the most suitable for the final formulation. Therefore, maceration was carried out for 60 days, with the mixture remaining in contact with the solid matrix. After this period, the alcoholic extracts were filtered using a cartridge filter with thread (5 × 2 1/2″, polypropylene thread and core), and inverted sugar was added at a concentration of 20%.The proportion of inverted sugar was determined to meet the standards established for liqueurs by national legislation (between 100 and 350 g⋅L^−1^ for fine liqueurs) [14] and other international regulations, including the Code of Federal Regulations [22] of the United States (a minimum of 2.5% sugar by volume) and the European Parliament and the Council of the European Union (100 g⋅L^−1^) [23]. Subsequently, the liqueurs were kept in a dark place at room temperature to be maturated for an additional seven days, underwent a second filtration, and were bottled for further analysis in 1 L glass containers with pre-sterilized caps.

### 3.4. Physicochemical Characterization of the Liqueur

The yield of the liqueurs was determined according to Equation (1).

Y = (Initial mass (g) ⋅ Final mass (g)^−1^) ⋅ 100 (1)

To evaluate the compliance of the produced liqueurs with the identity and quality standards established by national legislation [14], the analyses of alcohol content, total sugar determination, methanol content, and metallic contaminants were performed. The alcohol content (% by volume) was determined according to the methodology described by the Adolfo Lutz Institute [48]. To estimate the actual alcohol content of the finished liqueurs, aliquots (100 mL) of each liqueur were subjected to distillation in a fractional distiller with a heating mantle (102E, Fisatom, São Paulo, Brazil). The alcohol content of the distillate was determined using a densitometer (Density Meter DMA 4500M, Anton Parr, Graz, Austria), which converts the liquid density into alcohol content based on reference tables or the internal equations of the equipment. Determining total sugars in the liqueurs was performed according to the Lane–Eynon methodology [48].

The determination of methanol content in the liqueurs was performed via gas chromatography with flame ionization detection (GC-FID) (Gas Chromatograph, Clarus 480, PerkinElmer, Shelton, CT, USA), using the sample distillate spiked with an internal standard (methyl-4-pentanol-2). The separation was carried out in a Carbowax 400 column at 5% and Hallcomid M.18 OL at 1%, coated on Chromosorb W (60–80 mesh) inside a stainless steel column, 7.5 m in length and 1/8″ in diameter. The chromatographic support (Chromosorb W) was pre-activated in an oven at 750–800 °C for 4 h, as recommended by the International Organisation of Vine and Wine [49].

The determination of metallic contaminants (lead and copper) was performed with atomic absorption spectrometry, following the methodologies described in the Standard Methods for the Examination of Water and Wastewater (Methods 3120B and 3111B, respectively) [50].

For the physicochemical characterization of the produced liqueurs, duplicate analyses were performed for the following parameters: SS, pH [48], and TA [51]. Additionally, the TPC [52] and chromaticity parameters [53] were determined. A benchtop spectrophotometer (ColorFlex45/0 model, Hunterlab, Reston, VA, USA) was used for the determination, with D65 illuminant settings and a 10° observer angle. The Universal Software version 4.10 was used to determine the absolute values of L* (ranging from 0 to 100, from black to white), a* (variation between green and red), and b* (variation between blue and yellow). The color specification system used was CIELAB, which utilizes the CIE L*, a*, b* scale, defined by the Commission Internationale de l’Éclairage (CIE). Using the absolute values (a* and b*), C* was calculated in cylindrical coordinates through Equation (2), and the h° was determined using Equations (3), (4) or (5), depending on the values of a* and b* [31,54]. The CIRG was calculated using Equation (6) [55].

C = (a*^2^ + b*^2^)^0.5^ (2)

h° = tan^−1^ (b* ⋅ a*^−1^), where + a* and + b* (3)

h° = 180 + tan^−1^ (b* ⋅ a*^−1^), where − a* and + b* (4)

h° = 360 + tan^−1^ (b* ⋅ a*^−1^), where − a* and − b* (5)

CIRG = (180-h°)/(L* + C*) (6)

### 3.5. Identification and Quantification of Anthocyanins

The composition of anthocyanins in BRS Carmem grape liqueurs was determined via high-performance liquid chromatography coupled with a diode array detector and mass spectrometry with electrospray ionization (HPLC-DAD-ESI-MS^n^), without the need for prior extraction. To remove excess sugars and acids present in the liqueurs, aliquots of 4 mL of the samples were subjected to solid-phase extraction (SPE) using SPE-C18 cartridges (Sep-Pak Plus, Waters^®^, Milford, CT, USA) containing 820 mg of adsorbent phase, according to the methodology described by Rebello et al. [56]. The partially purified liqueurs were then concentrated by rotary evaporation (Hei-VAP Precision, Heidolph, Schwabach, Germany), diluted in 0.1 N HCl, and filtered through polyester membranes (0.20 μm, Chromafil PET 20/25, Macherey-Nagel, Düren, Germany) before injection into the chromatographic system.

For the chromatographic analyses, 10 µL aliquots were injected into a chromatographic system consisting of an Agilent 1100 series chromatograph (Agilent, Waldbronn, Germany), equipped with a diode array detector (DAD) (Model G1315B, Agilent, Germany) and coupled to an ion trap analyzer (LC/MSD Trap VL, G2445C VL) through an electrospray ionization system (ESI-MSn). During the analysis, a C18 reverse-phase chromatographic column (Zorbax Eclipse XDB, 2.1 × 150 mm, 3.5 µm particle, Agilent) was maintained at a temperature of 40 °C. All sample preparation and injection steps were performed in duplicate for each biological replicate.

The mobile phase consisted of the following solvents: (A) acetonitrile/water/formic acid (3:88.5:8.5, *v*/*v*/*v*) and (B) acetonitrile/water/formic acid (50:41.5:8.5, *v*/*v*/*v*). The linear gradient program was as follows: 0 min, 94% A and 6% B; 10 min, 70% A and 30% B; 30 min, 50% A and 50% B; 34 min, 100% B; 36 min, 100% B; 42 min, 96% A and 4% B; and 50 min, 96% A and 4% B. The ESI-MS^n^ ion trap analyzer was operated in positive mode, following the parameters established by Rebello et al. [56]: drying gas (N2) at 8 L⋅min^−1^, drying temperature of 325 °C, and nebulizer (N2) at 50 psi. Ionization and fragmentation settings were optimized through direct infusion of appropriate standard solutions (mv-3-glc and mv-3.5-glc in positive ionization mode), with a scanning range of 50–1200 *m*/*z*.

The identification of compounds was based primarily on UV–Vis and MS/MS spectroscopic data obtained from analytical standards or previous studies [56,57,58,59]. The identified anthocyanins were presented qualitatively as the molar ratios (%), normalized to the total anthocyanin content. For quantification, compounds extracted from the DAD chromatograms at 520 nm were quantified using standard calibration curves of mv-3-glc and mv-3.5-glc to express the total anthocyanin content in two ways: mg equivalents of mv-3-glc⋅kg^−1^ and mv-3.5-glc⋅kg^−1^, respectively.

### 3.6. Sensory Analysis

The characterization of the liqueurs was performed through affective sensory analysis, including acceptance tests and purchase intention, with the goal of assessing the influence of the alcoholic source used in the formulation (A or C). This study was approved by the Ethics and Research Committee of the Institute of Biological Sciences, Letters, and Exact Sciences (IBILCE) of the São Paulo State University “Júlio de Mesquita Filho”, Unesp, São José do Rio Preto Campus, Brazil, under CAAE nº 78781324.5.0000.5466. All experimental activities were conducted in the sensory analysis laboratory located on the aforementioned campus, in individual booths previously sanitized and under direct white lighting.

Before participating in the sensory analysis, the evaluators provided written informed consent and filled out a characterization form with information regarding their preference and frequency of liqueur consumption. Only participants considered potential consumers were included in the analysis. These individuals were defined as those aged 18 years or older, regardless of gender, who indicated that they liked liqueurs or consumed them at least once every six months.

For the sensory tests, the samples were presented in transparent acrylic cups (40 mL), previously coded with random three-digit numbers, containing 15 mL of each liqueur (A or C). Samples of both formulations were offered in a monadic and balanced manner [60]. Consumers were asked to evaluate the samples based on the degree of liking for eight attributes: appearance, color, consistency, aroma, flavor, sweetness, alcohol content, and overall impression, using a balanced nine-point hedonic scale: 9—extremely liked; 8—liked very much; 7—moderately liked; 6—slightly liked; 5—neither liked nor disliked; 4—slightly disliked; 3—moderately disliked; 2—disliked very much; 1—extremely disliked. Finally, consumers were asked about their purchase intention using a five-point balanced scale (1—would definitely not buy; 5—would definitely buy).

### 3.7. Statistical Analysis

All results were expressed as mean ± standard deviation. For the comparison of physicochemical results, Student’s *t*-test was performed using Microsoft Excel (Microsoft Corporation, Redmond, WA, USA), which was also used to generate frequency distribution graphs for purchase intention data from the sensory acceptance test. The acceptance scores were subjected to a two-way analysis of variance (ANOVA), with samples and consumers as factors, followed by Tukey’s test (α = 0.05) using Statistica 10.0 (StatSoft Inc., Tulsa, OK, USA). The Pearson correlation test and the radar chart were conducted in the R environment (4.1.3) [61] using the “corrplot (version 0.95)” [62] and “fmsb (version 0.7.6)” [63] packages, respectively.

## 4. Conclusions

The two types of liqueurs produced showed physicochemical properties with significant differences caused by the alcoholic source. The liqueur made with cereal alcohol exhibited higher values of soluble solids, acidity, and total phenolic content (TPC) compared to the one made with cachaça, in addition to greater color saturation (C*) and lower lightness (L*). However, despite these physicochemical differences, the sensory analysis revealed no significant difference in overall acceptance or purchase intention between the two liqueurs, indicating no clear consumer preference. The cereal alcohol-based liqueur stood out in terms of appearance and color, while the cachaça-based liqueur was superior in aroma. Thus, the use of cereal alcohol may be more suitable for producers aiming to enhance the fruit color in the final product. At the same time, further analysis of volatile compounds is recommended to understand better the actual influence of cachaça-derived volatiles on the liqueur’s aromatic profile. Both liqueurs presented an interesting anthocyanin profile. The complexity of the anthocyanins identified may contribute to greater pigment and color stability during storage, warranting further investigation in future studies. The data obtained may contribute to expanding the scientific literature on anthocyanin composition in more innovative alcoholic beverages. It can be concluded that, despite differences in the alcohol source, both grape liqueurs represent an excellent alternative for the utilization of surplus fruit production and the generation of additional income for producers.

## Figures and Tables

**Table 1:** Physicochemical characterization of BRS Carmem grape liqueurs produced with different alcoholic sources.

Parameter ^1^	Cereal Alcohol	White Cachaça
SS (°Brix)	23.25 ± 0.01 *	19.50 ± 0.01
Total sugar content (g⋅L^−1^)	177.3 ± 6.9 *	149.5 ± 2.0
TA (g tartaric acid⋅100 g^−1^)	0.39 ± 0.06 *	0.33 ± 0.04
pH	2.77 ± 0.06	2.87 ± 0.02 *
TPC (mg gallic acid⋅100 g^−1^)	607.45 ± 27.83 *	457.64 ± 11.86
Lightness (L*)	26.51 ± 0.63	30.64 ± 0.25 *
Chromaticity (C*)	29.38 ± 0.52 *	24.55 ± 0.31
Hue angle *(h°*)	59.16 ± 1.77	63.18 ± 2.34 *
Color Index for Red Grapes (CIRG)	2.16 ± 0.10	2.12 ± 0.08
Alcohol content (% *v*/*v* at 20 °C)	21.59 ± 1.49	20.14 ± 0.28
Methyl alcohol (mg⋅100 mL^−1^)	18.65 ± 2.09 *	10.47 ± 1.33
Copper (mg⋅kg^−1^)	<0.3	<0.3
Lead (mg⋅L^−1^)	<0.1	<0.1

^1^ SS: soluble solids, TA: total acidity, TPC: total phenolic compounds. * in the same row indicates the highest significant mean (*p* ≤ 0.05) between samples, as determined with Student’s *t*-test.

**Table 2:** Anthocyanin profile of BRS Carmem liqueurs obtained from different alcohol sources analyzed with HPLC-DAD-ESI-MS/MS (positive ionization mode). Data expressed as mean ± standard deviation (*n* = 4).

Anthocyanin ^1^	Molecular Ion; Product (*m/z*)	Molar Ratio	
		Cereal Alcohol	White Cachaça
Diglycosylated (3,5-glc)			
dp-3-cmglc-5-glc	773; 611, 303	0.62 ± 0.16	0.50 ± 0.21
cy-3,5-glc	611; 449, 287	0.40 ± 0.02	0.40 ± 0.03
cy-3-cmglc-5-glc	757; 595, 287	0.34 ± 0.09	0.34 ± 0.03
pt-3,5-glc	641; 479, 317	0.60 ± 0.12	0.51 ± 0.14
pn-3,5-glc	625; 463, 301	8.17 ± 0.85	8.52 ±1.08
pn-3-cfglc-5-glc	787; 625, 301	1.25 ± 0.13	1.16 ± 0.03
pn-3-cmglc-5-glc	771; 609, 301	3.60 ± 0.21	3.44 ± 0.15
mv-3,5-glc	655; 493, 331	36.03 ± 2.91	38.36 ± 2.74
mv-3-cfglc-5-glc	817; 655, 331	12.88 ± 0.27	12.79 ±0.21
mv-3-cmglc-5-glc	801; 639, 331	33.81 ± 2.86	31.89 ± 3.02
Monoglycosylated (3-glc)			
pn-3-glc	463, 301	0.71 ± 0.06	0.70 ± 0.05
mv-3-glc	493, 331	1.27 ± 0.15	1.13 ± 0.13
mv-3-cmglc	639, 331	0.32 ± 0.08	0.25 ± 0.09
Ratio (3,5-glc/3-glc)		43.39 ± 5.26	47.34 ± 5.60
Total anthocyanin (mg mv-3-glc⋅L^−1^)		212.76 ± 25.72 ^ns^	225.95 ± 57.24 ^ns^
Total anthocyanin (mg mv-3.5-diglc⋅L^−1^)		420.04 ± 16.39 ^ns^	456.44 ± 45.96 ^ns^

^1^ dp: delphinidin; cy: cyanidin; pt: petunidin; pn: peonidin; mv: malvidin; glc: glucoside; cfglc: 6″-(*p*-caffeoyl)glucoside; cmglc: 6″-(*p*-coumaroyl)glucoside. ^ns^ No significant differences between samples according to Student’s *t*-test (*p* > 0.05).

**Table 3:** Results for each attribute obtained in the sensory acceptance analysis of BRS Carmem grape liqueur obtained from different alcohol sources.

Radar Chart	Attribute	Cereal Alcohol	White Cachaça
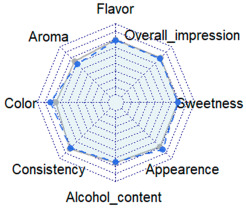	Appearance	7.6 ± 1.4 *	7.1 ± 1.6
	Color	7.5 ± 1.6 *	6.9 ± 1.7
	Consistency	7.3 ± 1.6	7.4 ± 1.4
	Aroma	6.2 ± 1.8	6.7 ± 1.7 *
	Flavor	7.1 ± 1.4	7.2 ± 1.6
	Sweetness	7.1 ± 1.6	7.1 ± 1.6
	Alcohol content	6.8 ± 1.8	6.9 ± 1.9
	Overall impression	7.1 ± 1.3	7.2 ± 1.4

* in the same row indicates the highest significant mean (*p* ≤ 0.05) between samples, as determined via two-way ANOVA (factors: sample × consumer). Radar chart: the blue area corresponds to the liqueur made with cereal alcohol, while the gray area represents the liqueur made with cachaça.

## Data Availability

The original contributions presented in this study are included in the article. Further inquiries can be directed to the corresponding author.

## Abbreviations

The following abbreviations are used in this manuscript:

A	Cereal alcohol
C	White cachaça
PC	Phenolic compound
SS	Soluble solids
TA	Total acidity
TPC	Total phenolic compounds
CIRG	Color Index for Red Grapes
mv	Malvidin
pn	Peonidin
pt	Petunidin
dp	Delphinidin
cy	Cyanidin
glc	Glucoside
cmglc	Coumaroil-glucoside
cfglc	Caffeoyl-glucoside
